# Mesenchymal Stem Cells and Cutaneous Wound Healing: Current Evidence and Future Potential

**DOI:** 10.1155/2015/831095

**Published:** 2015-05-27

**Authors:** M. Isakson, C. de Blacam, D. Whelan, A. McArdle, A. J. P. Clover

**Affiliations:** ^1^Department of Plastic and Reconstructive Surgery, Cork University Hospital, Cork, Ireland; ^2^Centre for Research in Vascular Biology, University College Cork, Cork, Ireland

## Abstract

Human skin is a remarkable organ that sustains insult and injury throughout life. The ability of skin to expeditiously repair wounds is paramount to survival. With an aging global population, coupled with a rise in the prevalence of conditions such as diabetes, chronic wounds represent a significant biomedical burden. Mesenchymal stem cells (MSC), a progenitor cell population of the mesoderm lineage, have been shown to be significant mediators in inflammatory environments. Preclinical studies of MSC in various animal wound healing models point towards a putative therapy. This review examines the body of evidence suggesting that MSC accelerate wound healing in both clinical and preclinical studies and also the possible mechanisms controlling its efficacy. The delivery of a cellular therapy to the masses presents many challenges from a safety, ethical, and regulatory point of view. Some of the issues surrounding the introduction of MSC as a medicinal product are also delineated in this review.

## 1. Introduction

Chronic wounds represent a significant biomedical burden. In the USA, more than 6 million Americans are affected by chronic wounds, with an annual cost estimated at $25 billion annually [1]. In Europe, almost 2% of health budgets are spent on managing chronic wounds [2].

Wound healing is an elaborate process that occurs in three distinct, yet overlapping, phases: inflammation, cell proliferation, and remodeling [3]. Adult cutaneous wound repair is characterized by a highly evolved fibroproliferative response to injury that quickly restores the skin barrier, thereby reducing the risk of infection and further injury. The inflammatory phase is characterized by influx of polymorphonuclear cells followed by monocytes/macrophages. Macrophages secrete the growth factors and cytokines necessary for wound healing such as interleukins, TGF-*β*, and tumor necrosis factors. Stimulated by these growth factors, healing proceeds to the proliferative phase, made up of fibroplasia, matrix deposition, angiogenesis, and reepithelialization. Remodeling is a dynamic phase during which various collagens are continuously deposited and degraded [4, 5].

Chronic wounds occur when there is a failure of injured skin to proceed through an orderly and timely process to produce anatomic and functional integrity. Causative factors include malnutrition and immunosuppression, and chronic wounds are commonly seen as a consequence of diabetes mellitus and vascular compromise. Current techniques to manage chronic wounds typically focus on modification of controllable causative factors: antibiotic treatment of infected wounds, pressure relief of decubitus areas, revascularization of ischemic limbs, and compression garments for venous insufficiency [6, 7]. Surgical debridement and negative pressure wound therapy are commonly employed techniques but remain a suboptimal treatment due to the lengthy healing time required for this method. The advent of skin substitutes has increased our armamentarium for treating this difficult condition, but to date no ideal therapy is available to treat troublesome, chronic wounds. Despite huge advances in medical care and nutrition which have resulted in a commendable change in the outcome of chronic wound management, new therapies in this area are required to optimize outcomes for our patients. Stem cells, with their unique properties to self-renew and undergo differentiation, are emerging as a promising candidate for cell-based therapy for the treatment of chronic wounds.

**Figure 1:**
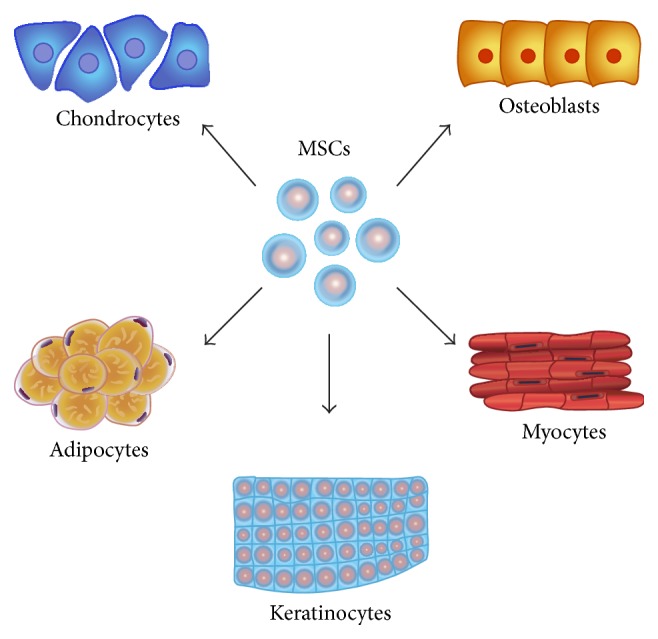
Multilineage potential of MSCs (mesenchymal stem cells), such as bone-marrow-derived mesenchymal stromal cells and adipose-derived stromal cells, has the potential to differentiate into various lineages, making them ideal candidates for cell-based tissue engineering strategies. It has been demonstrated that MSCs can undergo osteogenesis, chondrogenesis, adipogenesis, and myogenesis.

The term “stem cell” refers to a myriad of different cell types that share two key characteristics: self-renewal and the potential for differentiation into different cell types. Adult stem cells are not limited to the same extent by low availability and ethical concerns that limit the use of embryonic stem cells, thus making them an ideal cell type for tissue regeneration applications. Of the numerous types of adult stem cells that have been described in the literature, two types are of particular relevance to tissue engineering in the setting of chronic wounds: bone-marrow-derived mesenchymal stem cells (BM-MSCs) and adipose-derived stromal cells (ASCs). Bone marrow was the first source that was reported to contain MSCs [8]. However, the isolation of these cells is fraught with considerable donor site morbidity and low cell yield. ASCs represent a similar cell type of multilineage potential. They are isolated from the stromal vascular fraction of adipose tissue after a digestion and centrifugation step [9]. In 2001, Zuk et al. demonstrated that human fat obtained from human lipoaspirates contained multilineage stem cells, which have since been shown to have the potential to undergo adipogenesis, osteogenesis, chondrogenesis, and myogenesis* in vitro* and* in vivo* [10–14] (Figure 1).

Stem cells offer enormous potential for enhancing tissue repair and regeneration following injury. The rapidly developing fields of stem cell biology and skin tissue engineering have created translational opportunities for the development of novel stem cell-based wound healing therapies that show promising results in preclinical and clinical trials for the treatment of chronic wounds. In this review, we evaluate the current evidence for adult stem cell-based therapies and their application to chronic wound healing.

## 2. Methods

### 2.1. Data Sources and Searches

The topic “mesenchymal stem cells” AND “cutaneous wound healing” was explored to determine significant issues (conceptual mapping). From this, a search strategy was devised using the following key terms: (mesenchymal stem cells OR MSC OR stromal OR adipose derived) AND (cutaneous wound healing OR wound repair OR burn). Using these key terms, an electronic bibliographic search was conducted in MEDLINE and CENTRAL (The Cochrane Central Register of Controlled Trials) from inception until November 31, 2012. Limits were placed on each search to exclude non-English citations. Reference lists of all relevant publications were searched for additional papers. Hand searching of key journals was undertaken and relevant conference proceedings were also examined.

### 2.2. Study Selection

Inclusion and exclusion criteria for selection are listed in Table 1. A staged review of article titles and abstracts was performed to select all studies that met the inclusion criteria. Studies whose abstracts met the inclusion criteria were retrieved and the full text was analyzed. Papers, using animal models, selected for full text review are listed in Table 2.

### 2.3. Data Extraction, Synthesis, and Analysis

**Figure 2:**
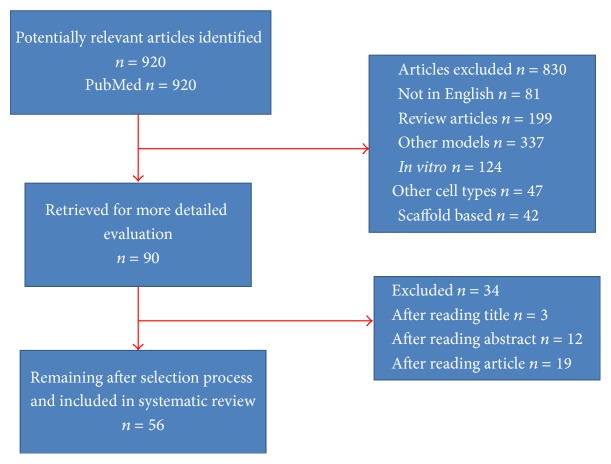
Prisma flowchart for systematic review. Flowchart demonstrating the selection criteria for research papers included in this review. Overall 56 papers were evaluated in this systematic review, including 50 using animal models and 6 using human trials.

Data extraction and quality assessment were performed, with the following variables being recorded from each study: cell source, characterization technique, recipient, injury model, cell delivery technique, and wound healing outcome. The papers included in our final analysis were heterogeneous in their methodology and results, thus precluding a formal pooled analysis (Figure 2). Therefore, a narrative summary of results was undertaken.

## 3. Results and Discussion

MSCs are nonhematopoietic stromal cells capable of multilineage differentiation that show enormous potential for clinical translation for the treatment of chronic wounds. Adult MSCs have been isolated from various sites, including bone marrow, adipose tissue, and amniotic fluid. Use of adult MSCs provides an easily accessible source of multipotent precursor cells that could potentially avoid the ethical concerns associated with other stem cell types, particularly embryonic stem cells. Additionally, transplantation with postnatal stem cells bypasses the possibility of immune rejection that could occur with other cell types [65].

ASCs and BM-MSCs share many common characteristics, including multilineage differentiation potential (Figure 1), morphology, telomerase activity, and gene expression [14]. In addition, they share a similar cell surface marker phenotype; however, a definitive profile that allows for the prospective isolation of MSCs has not been firmly established. In general, ASCs are considered to be CD45−CD235a−CD31−CD34+, with the addition of positivity for CD106 and CD36 distinguishing them from BM-MSCs [66, 67]; however, we found a variety of cell surface markers used to define these cells in the papers we examined.

### 3.1. Cutaneous Wound Models and Cell Processing Technique

**Figure 3:**
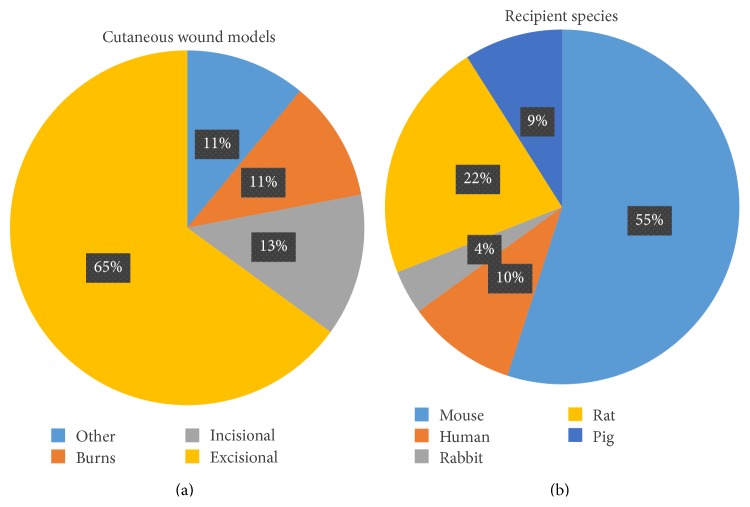
Studies in cutaneous wound healing were performed in a diverse range of animal models. Animal models used for cutaneous wound healing studies are not standardized. (a) Most studies of cutaneous wound healing were performed using excisional wound models in 65% of studies. Other models included incisional wounds and burn models. (b) Most studies were carried out in mouse models (55% of studies) with other models including rat, human, pig, and rabbit models.

Rodent models were used in 78% of the papers assessed, with incisional, excisional, and burn wounds used to assess healing (Figure 3). The majority of studies measured healing based on gross examination of wound area. Rodents are typically used as animal models for preclinical wound healing studies. Rodents are attractive candidates for wound healing studies because of their availability, low cost, and ease of handling. However, rodent models have been criticized because the major mechanism of wound closure is contraction, whereas in humans reepithelialization and granulation tissue formation are the major mechanisms involved [68]. The advent of a novel wound splinting model, utilizing a silicone splint in rodents, has allowed for an accurate, reproducible model of wound healing that facilitates “humanized” wound healing through the processes of granulation and reepithelialization [68].

Thirty-five articles were identified that harvested BM-MSCs and 22 that used ASCs. One of the drawbacks to direct comparison between papers was the lack of uniform cell isolation and delivery methods used in the studies. In terms of cell isolation, studies employed various techniques using either freshly isolated cells, with or without prospective isolation for subpopulations using flow cytometry, or periods of* in vitro* expansion. The lack of a standard cell profile phenotype and standard cell isolation protocol is one of the major limitations that is hindering translation of adult stem cell therapies. Of note, adult stem cells represent a heterogeneous cell population and interestingly subpopulations within these compartments are absent in certain conditions, such as diabetes, in which functional stem cells are required to adequately treat conditions [69, 70]. Additionally, it is important to note that cell surface phenotype can undergo significant phenotypic drift following a period of* in vitro* culture expansion and thus alter the ability to prospectively isolate multipotential subpopulations, perhaps altering their differentiation potential of cells* in vivo* [71]. Therefore, it is important that standardized protocols are developed to allow for clinical translation of these studies.

In addition to variations in cell isolation techniques, cell delivery methods for adult stem cells are also nonstandardized in the literature. Both topical and systemic delivery methods have been shown to be effective. For the purposes of this review, we considered topical, intradermal, and subcutaneous delivery as “local” and intramuscular, parabiotic, and intravenous delivery as “systemic” (Table 2). Local application with syringe-spray systems is the approach being utilized in current clinical trials [72, 73]. It is important to know that the choice of biomimetic scaffold plays a pivotal role in driving appropriate tissue regeneration* in vivo*. Since the extracellular matrix (ECM) plays a key role in guiding various biological processes, creating a scaffold that mimics the normal ECM should enhance tissue regeneration. As a result, advances in bioengineering have resulted in a myriad of cell delivery mechanisms and various scaffolds that again make comparison between methods difficult [55, 74, 75]. The local environment, including neighboring cells, soluble signaling molecules, ECM, mechanical forces, oxygen tension, and other factors, is crucial to enable stem cells to maintain their regenerative potential [76]. Ongoing research continues to identify novel techniques to deliver cellular therapeutics to specific locations in order to enhance cell survival and function, in what is typically a hostile wound environment [77, 78].

### 3.2. Wound Healing Results

All of the articles included in this study evaluated time to healing using adult stem cells in wound healing. All of studies demonstrated accelerated wound closure and enhanced histological parameters in wounds treated with MSC therapies, irrespective of cell isolation or delivery method.

Despite a lack of a standardized histological scale for evaluating cutaneous wound healing, collagen deposition, neovascularization, and cellular infiltration are considered representative features. All studies that reported histological findings identified enhanced wound healing in wounds treated with MSCs compared to control treatment [17, 31, 47, 55, 57, 79, 80]. Specific features observed included increased recruitment of macrophages [21], increased angiogenesis [55, 62], and restoration of sebaceous glands and hair follicles [55].

A number of studies sought to evaluate the persistence of MSCs in the wound environment after transplantation and demonstrate that MSCs persist in the wound for up to several weeks following transplantation [17, 31, 33, 44]. Various mechanisms have been investigated to enhance cell survival following transplantation and typically involve alterations in biomimetic scaffold delivery systems [55].

### 3.3. Preclinical Studies

The majority of studies evaluating the role of MSCs in wound healing have been carried out in preclinical animal models. Exogenous application of MSCs during injury repair has been shown to be therapeutic in animal models. Several studies examining local injection of murine MSCs into a mouse model of incisional full thickness wound healing have consistently shown accelerated time to wound closure [21, 62, 81] with increased angiogenesis, reepithelialization, and recruitment of myeloid cells into the wound. Genetic manipulation of MSCs to overcome the hostile wound environment is emerging as a novel technique to enhance cell survival and proliferation, ultimately accelerating wound healing in animal models [82]. This is particularly important in the setting of diabetic and vascular wounds, in which local cytokine levels are inadequate to achieve normal wound healing.

Systemic delivery of adult stem cells has also been demonstrated to accelerate wound healing in cutaneous wound models. Once-daily administration of 2 × 10^6^ cells over 4 days after wounding resulted in significantly increased wound breaking strength at days 7 and 14 [49]. In one study, MSC-treated wounds regained 52% of normal dermal tensile strength while untreated controls regained 31% of tensile strength compared to unwounded skin at 80 days following transplantation [17].

While systemic delivery of MSCs has been shown to deposit cells at the injury site, cell engraftment and survival have been limited. This has led to the majority of studies looking at novel mechanisms to locally deliver adult stem cells to enhance wound healing. Ultimately, this has generated significant collaborations between stem cell researchers and bioengineers and has resulted in the emergence of a plethora of cell delivery systems to enhance wound healing. Ideally, to identify the best strategy for cell delivery, direct comparison of various cell delivery systems is required.

### 3.4. Clinical Studies

Currently, there are four published clinical studies using MSC in cutaneous wound healing [24, 83–85] and a handful of single case studies [86]. Preclinical and early human trials identified in this review demonstrated that MSCs accelerated wound closure, increased tensile strength, and promoted cytokine production and angiogenesis. In 2007, Falanga et al. demonstrated accelerated healing of acute surgical wounds in human subjects (*n* = 5) when treated with BM-MSC delivered in a fibrin spray [24]. Wounds were biopsied and histology suggested that at least some MSCs migrated into the upper layers of the wound bed and differentiated into a fibroblast phenotype. Chronic venous and diabetic ulcer wounds were also examined (*n* = 6) and a significant decrease in size at 16 weeks following three topical applications of MSCs was observed.

The first randomized study in humans was produced by Dash et al. in 2009, who compared intramuscular/subcutaneous injection of BM-MSCs to standard wound care in chronic nonhealing wounds [84]. A significant decrease in ulcer size was observed in the treated group. Yoshikawa et al. introduced MSCs impregnated onto a collagen sponge topically to 20 chronic wounds and recorded complete closure in 13 cases [87]. Another case series examined 3 patients with chronic cutaneous ulcerations [83]. In this study, patients received local BM aspirate in addition to 3 additional treatments with cultured BM-MSC. All patients showed clinical improvement in their wounds within days following administration of bone marrow aspirate or cultured bone marrow cells. Wounds showed a steady overall decrease in wound size, and an increase in the vascularity of the dermis and in the dermal thickness of the wound bed was histologically suggested. No adverse events related to the delivery of bone marrow aspirate or the cultured cells were noted [83]. Arising from these early clinical studies, several trials are currently recruiting, which will examine long-term efficacy of BM-MSC therapy on diabetic and venous ulcers [73, 88, 89].

### 3.5. Mechanism of Action

**Figure 4:**
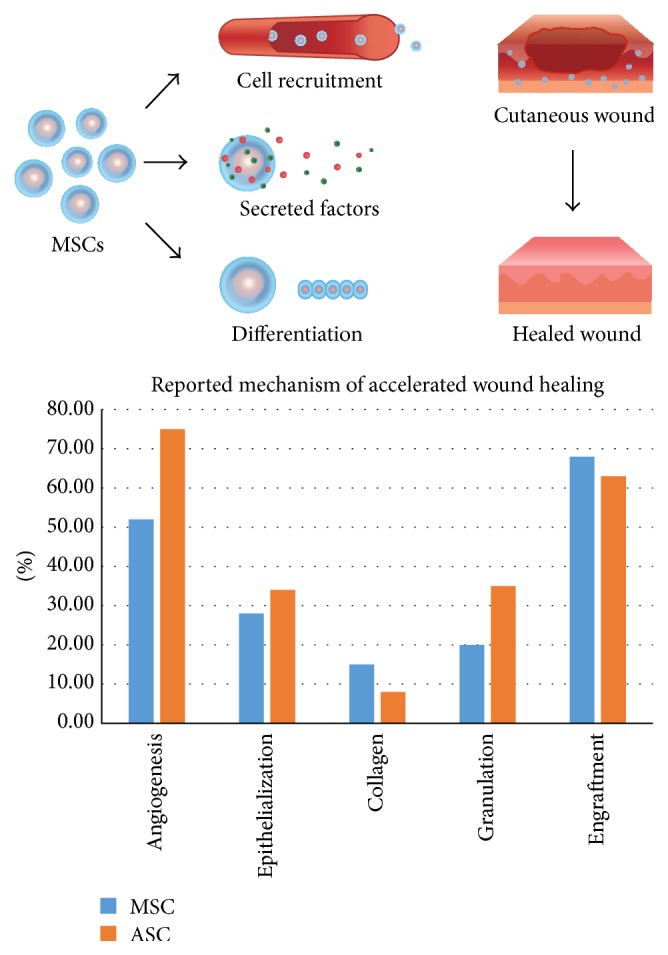
MSCs and ASCs act to promote cutaneous wound healing through a variety of mechanisms. MSCs and ASCs influence wound healing through a variety of mechanisms, including angiogenesis, promoting epithelialization, and enhancing collagen deposition and granulation tissue formation. In addition, various studies have demonstrated that transplanted cells engraft into the wound to participate in wound healing. Blue bars represent studies examining BM-MSCs and orange bars represent studies evaluating ASCs.

The true mechanism of action of MSCs in accelerating wound closure is not fully understood. The current thinking is that MSCs can enhance wound healing through two main mechanisms: by providing the necessary cues for wound healing through the release of inflammatory mediators, together with key cytokines and growth factors, in addition to the cells themselves participating in the process of wound healing, ultimately differentiating into the cell types required for closure of the wound (Figure 4).

Studies carried out* in vitro* and* in vivo* studies have demonstrated that transplanted MSCs can differentiate into cells of the residing tissue, repair damaged tissue, and at least partially restore its normal function [90]. Ma et al. demonstrated* in vitro* differentiation of MSCs into a multilayered epidermis-like structure which expressed the epidermal markers cytokeratin-10 and filaggrin [91]. In their clinical study, Sheng et al. demonstrated recovery of sweat gland function following MSC transplantation into excision wounds in rodent skin [57]. In addition to their differentiation capacity, increasing evidence points to the ability of MSCs to secrete paracrine factors that modulate the local environment and stimulate wound healing [92]. Specifically, MSCs have been shown to significantly decrease the production of proinflammatory cytokines in the acute period when high levels can be deleterious to tissue and to upregulate them in the later regeneration phase [93]. Protein arrays have demonstrated that conditioned media from MSC cultures contain various cytokines and chemokines such as IL-8, IL-6, TGF-*β*, and VEGF, all of which are essential to normal wound healing [94].

MSCs have been shown to enhance wound healing through increased angiogenesis, reepithelialization, and granulation tissue formation (Figure 4). MSCs express keratinocyte-specific markers and high levels of vascular endothelial growth factor and angiopoietin-1, suggesting that MSCs promote wound healing by differentiation and release of proangiogenic factors. Yet, other studies have demonstrated that intravenous (IV) injection in mice induced MSC transdifferentiation into keratinocytes, endothelial cells, and pericytes in cutaneous wounds. When human BM-MSCs were applied to full thickness skin defects in mice in conjunction with IV MSC administration, all wounds healed without a scar or retraction [95].

A recent hypothesis is that MSCs are pericytes, a supporting cell for blood vessels [65]. This hypothesis raises an interesting connection between these cells and angiogenesis, a key component in wound repair [65].

### 3.6. Safety and Regulation

Despite the rapid progress in evaluating the efficacy of MSCs in wound healing, many issues still need to be addressed. A lack of standardized isolation and delivery mechanisms for MSCs exists. Uncertainties remain as to how to best identify an ideal subpopulation of MSCs and whether freshly isolated cells are superior to cells that undergo a period of culture expansion* in vitro*.

All of these findings from animal transplantation studies demonstrate that MSCs can contribute to wound repair and may provide the cell source for regenerative therapy. However, further studies are necessary to extensively study not only MSCs but also the critical factors that make up the microenvironment that supports the survival and differentiation of these cells. This would allow us to determine the extent to which MSCs in the wound environment act as multipotent cells or a source of secreted factors. This research would also divide this heterogeneous cell type into more distinct and functional subpopulations.

**Figure 5:**
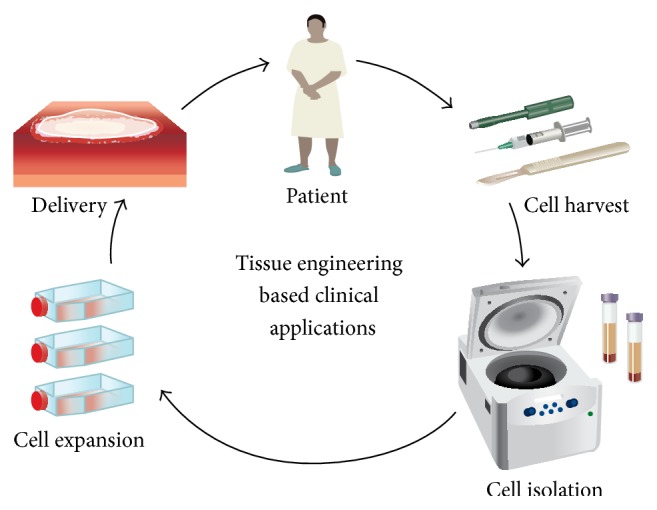
Work flow of cell-based regenerative therapy. An ideal regenerative medicine strategy requires three components: an ideal cell type, biomimetic scaffold, and factors to create the desired biological response* in vivo*. Cutaneous wounds represent a harsh environment for cellular therapy due to their hypoxic environment and low pH which can affect the survival and potential of transplanted cells. The niche microenvironment can be manipulated with the use of scaffolds and growth factors to enhance cellular survival, promote cellular differentiation, and ultimately enhance wound healing in the clinical setting. Reproduced with permission from the authors: McArdle, Paik, Chung, Hu, and Walmsley et al. (2013) Manipulation of Stem Cells and their Microenvironment for Tissue Engineering. Surgery Curr Res 3 : 134.

A major obstacle to clinical translation of cellular therapies is safety and regulation of their use. Safety concerns are apparent at all stages from isolation to administration (Figure 5 details the isolation workflow). Transitioning from preclinical research in terms of* in vivo* models to the clinical arena represents a major step. The manipulation of MSCs for therapeutic modalities must be done in accordance with good manufacturing practices and the regulations of the FDA and/or European Medicines Agency. Other obstacles that must be addressed include the need for development of suitable serum-free media for these cells as fetal bovine serum is not recommended for clinical therapies due to the risk of contamination and infection [96].

### 3.7. Future Directions

Other approaches have concentrated on the delivery of MSC transfected with genes or in a suspension with plasmids or growth factors such as ectodysplasin, basic fibroblast growth factor (bGFG), or human hepatocyte growth factor [97–100]. The loss of sweat glands and thermal regulation after severe thermal injury has been a problematic area in tissue regeneration due to the multiple germ layers involved. Transplanted MSC transfected with ectodysplasin, a gene implicated in the development of sweat gland structures, into scalded paws of mice was shown to be beneficial in sweat gland regeneration [97]. Each mouse received a full thickness burn on each posterior paw and after 30 mins received a subcutaneous injection of 1 × 10^6^ human BM-MSCs transfected with ectodysplasin. Treated animals expressed sweat gland phenotypes, cytokeratin-14, and carcinoembryonic antigen (CEA) and tested positive for perspiration [97].

As knowledge in tissue regeneration expands, researchers are exploring the synergistic effects of combining various approaches, such as augmenting cellular therapy, with other growth factors in combination with a delivery scaffold which can control the release rate of both the cells and factors into the wound [48, 50, 101].

## 4. Conclusion

Stem cells possess a distinct ability to self-renew and differentiate, making them a more attractive cell for cell-based therapies. Ethical concerns have limited the use of embryonic stem cells in regenerative medicine, and current focus for clinical translation lies with adult stem cells. Adult stem cells are an exciting source for wound healing applications, owing mostly to their relative ease of harvest and the ability to yield large quantities of cells. In this review, we identify that adult stem cells demonstrate huge promise in the treatment of chronic wounds. Studies evaluating the role of BM-MSCs and ASCs in treating chronic wounds demonstrate accelerated wound healing through a variety of mechanisms.

With every new scientific advancement, it is the responsibility of scientists and physicians to guide and educate the public on the advantages and disadvantages of any proposed therapy. Overstating potential benefits based on incomplete evidence can only serve to erode the public's trust in the medical profession and, more concerning, compromise the safety of our patients [102]. Currently, clinical translation of adult stem cells for the treatment of chronic wounds is hindered by a lack of standardized protocols for cell characterization, isolation, and transplantation.

Areas that deserved further attention include establishing a more comprehensive understanding of the signaling network that reliably leads to robust new tissue formation, together with the identification of a definitive cell surface marker profile.

Despite the current limitations to widespread clinical use, BM-MSCs and ASCs are highly promising cell sources for the treatment of chronic wounds.

## Figures and Tables

**Table 1:** Exclusion/inclusion criteria.

Inclusion criteria	Exclusion criteria
(1) Original scientific studies	(1) Review articles
(2) Studies investigating mesenchymal stem cells *in vivo *	(2) *In vitro* studies
(3) Studies involving cells derived from bone marrow or adipose tissue	(3) Studies involving noncutaneous models
(4) Studies involving cutaneous wounds or burns	(4) Studies involving radiation injury models

**Table 2:** Experimental parameters and reported mode of action of reviewed articles.

Author	Cell source	Wound model	Cell delivery method	Cell number	Mechanism of action
Altman et al. [15]	Human	Excisional	Seeded matrix	1 × 10^5^ seeded twice	Local engraftment with endothelial and fibroblastic phenotype
Altman et al. [16]	Human	Excisional	Silk fibrin chitosan scaffold	1 × 10^5^	N/A
Argôlo Neto et al. [17]	Mouse	Excisional	Unclear	N/A	↑ collagen types I & III
Blanton et al. [18]	Pig	Excisional	Fibrin spray matrix	18 × 10^5^	
Borena et al. [19]	Rabbit	Excisional	SC	1.4 × 10^8^	↓ inflammation, ↑ neovascularization, ↑ fibroblast number, ↑ collagen thickness
Chen et al. [20]	Mouse	Excisional	Intradermal and topical	1 × 10^6^	MSCs localised to wound bed and appendages on day 14
Chen et al. [21]	Mouse (CM)	Excisional	Intradermal and topical	N/A	Recruitment of macrophages and progenitor cells (CD34, c-kit, Flk-1)
Cho et al. [22]	Human (CM)	Excisional	Intradermal	N/A	Enhanced histology score at d7, d14
Ebrahimian et al. [23]	Not specified	Excisional	IV and IM	1 × 10^6^	↑ perfusion, ↑ angiogenesis, ↑ collagen content, ↑ VEGF
Falanga et al. [24]	Mouse	Excisional	Topical fibrin scaffold	1 × 10^6^/cm^2^	Engraftment of MSCs at d18
Fu et al. [25]	Pig	Burn	Fibrin mesh scaffold +/− cytokines	2 × 10^6^	↑ capillary density
Fu et al. [26]	Pig	Excisional	Topical	N/A	
Gao et al. [27]	Human (CM)	Ischemic flap	Local	N/A	↑ capillary density, perivascular engraftment, ↑ VEGF, ↑ HIF1-a expression
Gu et al. [28]	Human	Ischemic flap	Intradermal	1 × 10^5^	No differences in total blood vessel number
Hamou et al. [29]	Mouse	Ischemic flap	Systemic	N/A	MSCs localize to ischemic area and engraft locally
Heo et al. [30]	Human (CM)	Excisional	Topical (+/− TNF-a)	N/A	↑ capillary density, ↑ VEGF, ↑ bFGF
Hou et al. [31]	Human	Excisional	Collagen	2 × 10^4^	↑ capillary density
Huang et al. [32]	Mouse	Excisional	Topical microsphere scaffold	1 × 10^6^	↑ vascularity and cellularity in treatment groups
Javazon et al. [33]	Mouse	Excisional	Topical	7.5 × 10^5^	↑ epithelialization and enhanced granulation tissue formation
Kataoka et al. [34]	Mouse	Excisional	SC	5 × 10^6^	Cell engraftment in epidermis, hair follicles, sebaceous glands and dermis, blood vessels
Kim et al. [35]	Rat	Excisional	Collagen scaffold	2 × 10^6^	↑ neovascularization
Kim et al. [36]	Human	Excisional	Collagen scaffold	1 × 10^6^	Significant difference in skin structure and dermal inflammatory cell infiltrate
Kim et al. [37]	Canine	Excisional	Intradermal +/− low level laser therapy	1 × 10^6^	↑ epithelialization rate, ↑ granulation tissue thickness, ↑ capillary number
Kwon et al. [38]	Rat	Incisional	IV or intradermal	N/A	↑ neovascularization, ↑ collagen production, ↑ expression of VEGF, EGF, PDGF-BB, TGF-B
Lee et al. [39]	Mouse	Incisional	Unclear	5 × 10^4^	N/A
Lee et al. [40]	Human (CM)	Excisional	Collagen scaffold	N/A	N/A
Lee et al. [41]	Human	Excisional	Collagen gel	N/A	↑ blood vessel density
Li et al. [42]	Human	Excisional	IV	N/A	Local cell engraftment
Lim and Yoo [43]	Mouse	Excisional	Intradermal	0.6 × 10^6^	↑ fibroblast numbers
Lin et al. [44]	Human	Excisional	Cell sheet (1 or 3 layers)	1 × 10^6^/layer	↑ epithelialization
Liu et al. [45]	Pig	Burn	Topical (scaffold)	2 × 10^6^	↑ capillary density, ↑ keratinization
Liu et al. [46]	Mouse	Excisional	Scaffold matrix	1 × 10^5^ seeded twice	↑ vessel density
Maharlooei et al. [47]	Rat	Excisional	Intradermal	1 × 10^6^	↑ epithelialization, ↑ granulation, ↑ neovascularization
Mansilla et al. [48]	Rabbit	Burn	Fibrin mesh scaffold	2 × 10^6^/mL/cm^2^	Heavy mononuclear infiltrate
McFarlin et al. [49]	Rat	Incisional	IV or SC	6 × 10^6^	↑ collagen production, ↓ inflammation
Nakagawa et al. [50]	Human	Excisional	Collagen scaffold	5 × 10^6^	↑ epithelialization
Nambu et al. [51]	Mouse	Excisional	Seeded atelocollagen matrix	5 × 10^5^	N/A
Nambu et al. [52]	Rat	Excisional	Seeded atelocollagen matrix	1 × 10^6^	↑ capillary density, ↑ inflammatory infiltrate, ↑ cell proliferation
Nie et al. [53]	Rat	Excisional	Intradermal	1 × 10^6^	↑ capillary number, ↑ granulation thickness
Rasulov et al. [54]	Rat	Burn	Topical	2 × 10^4^	N/A
Rustad et al. [55]	Mouse	Excisional	Collagen hydrogel topical or SC	2.5 × 10^5^	↑ MCP-1, VEGF-a/b, FGF1, MMP8, MMP9
Sasaki et al. [56]	Mouse	Excisional	IV	1 × 10^6^	↑ MSC migration due to SLC/CCL21
Sheng et al. [57]	Human	Burn	SC or ADM	1 × 10^6^	N/A
Shumakov et al. [58]	Rat	Burn	Topical	2 × 10^6^	↑ blood vessel density
Stoff et al. [59]	Human	Incisional	Intradermal	N/A	Engraftment through d21 with improved histology score
Tian et al. [60]	Mouse	Excisional	Intradermal and topical	1 × 10^6^	↑ epithelialization, ↑ vascularization, ↑ granulation tissue
Volk et al. [61]	Rabbit	Ischemic.	SC	0.5 × 10^6^	↑ granulation tissue
Wu et al. [62]	Mouse	Excisional	Intradermal and topical	1 × 10^6^	↑ VEGF, epithelialization, cellularity, appendages, and ↑ histology scores
Yang et al. [63]	Rat	Ischemic flap	SC	4 × 10^6^	↑ blood vessel density; cell engraftment in dermal and perivascular locations
Yeum et al. [64]	Mouse	Excisional	Scaffold (small intestinal submuocosa)	1 × 10^6^	↑ EGF, FGF, VEGF, IL-1-b, IL-4, ↓ TNF-a

## References

[1] Maan Z. N., Januszyk M., Rennert R. C. (2014). Noncontact, low-frequency ultrasound therapy enhances neovascularization and wound healing in diabetic mice. *Plastic and Reconstructive Surgery*.

[2] Posnett J., Gottrup F., Lundgren H., Saal G. (2009). The resource impact of wounds on health-care providers in Europe. *Journal of Wound Care*.

[3] Hu M. S., Maan Z. N., Wu J. C. (2014). Tissue engineering and regenerative repair in wound healing. *Annals of Biomedical Engineering*.

[4] Singer A. J., Clark R. A. F. (1999). Cutaneous wound healing. *The New England Journal of Medicine*.

[5] Ilan N., Mahooti S., Madri J. A. (1998). Distinct signal transduction pathways are utilized during the tube formation and survival phases of in vitro angiogenesis. *Journal of Cell Science*.

[6] McInnes E., Bell-Syer S. E., Dumville J. C., Legood R., Cullum N. A. (2008). Support surfaces for pressure ulcer prevention. *Cochrane Database of Systematic Reviews*.

[7] O'Meara S., Cullum N., Nelson E. A., Dumville J. C. (2012). Compression for venous leg ulcers. *Cochrane Database of Systematic Reviews*.

[8] Pittenger M. F., Mackay A. M., Beck S. C. (1999). Multilineage potential of adult human mesenchymal stem cells. *Science*.

[9] McArdle A., Chung M. T., Paik K. J. (2014). Positive selection for bone morphogenetic protein receptor type-IB promotes differentiation and specification of human adipose-derived stromal cells toward an osteogenic lineage. *Tissue Engineering Part A*.

[10] Zuk P. A., Zhu M., Ashjian P. (2002). Human adipose tissue is a source of multipotent stem cells. *Molecular Biology of the Cell*.

[11] Zuk P. A., Zhu M., Mizuno H. (2001). Multilineage cells from human adipose tissue: implications for cell-based therapies. *Tissue Engineering*.

[12] Cowan C. M., Shi Y.-Y., Aalami O. O. (2004). Adipose-derived adult stromal cells heal critical-size mouse calvarial defects. *Nature Biotechnology*.

[13] Zuk P. A. (2008). Tissue engineering craniofacial defects with adult stem cells? Are we ready yet?. *Pediatric Research*.

[14] Gimble J. M., Katz A. J., Bunnell B. A. (2007). Adipose-derived stem cells for regenerative medicine. *Circulation Research*.

[15] Altman A. M., Matthias N., Yan Y. (2008). Dermal matrix as a carrier for in vivo delivery of human adipose-derived stem cells. *Biomaterials*.

[16] Altman A. M., Yan Y., Matthias N. (2009). IFATS collection: human adipose-derived stem cells seeded on a silk fibroin-chitosan scaffold enhance wound repair in a murine soft tissue injury model. *Stem Cells*.

[17] Argôlo Neto N. M., Del Carlo R. J., Monteiro B. S. (2012). Role of autologous mesenchymal stem cells associated with platelet-rich plasma on healing of cutaneous wounds in diabetic mice. *Clinical and Experimental Dermatology*.

[18] Blanton M. W., Hadad I., Johnstone B. H. (2009). Adipose stromal cells and platelet-rich plasma therapies synergistically increase revascularization during wound healing. *Plastic and Reconstructive Surgery*.

[19] Borena B. M., Pawde A. M., Aithal H. P., Kinjavdekar P., Singh R., Kumar D. (2010). Evaluation of autologous bone marrow-derived nucleated cells for healing of full-thickness skin wounds in rabbits. *International Wound Journal*.

[20] Chen L., Tredget E. E., Liu C., Wu Y. (2009). Analysis of allogenicity of mesenchymal stem cells in engraftment and wound healing in mice. *PLoS ONE*.

[21] Chen L., Tredget E. E., Wu P. Y. G., Wu Y. (2008). Paracrine factors of mesenchymal stem cells recruit macrophages and endothelial lineage cells and enhance wound healing. *PLoS ONE*.

[22] Cho J.-W., Kang M.-C., Lee K.-S. (2010). TGF-*β*1-treated ADSCs-CM promotes expression of type I collagen and MMP-1, migration of human skin fibroblasts, and wound healing in vitro and in vivo. *International Journal of Molecular Medicine*.

[23] Ebrahimian T. G., Pouzoulet F., Squiban C. (2009). Cell therapy based on adipose tissue-derived stromal cells promotes physiological and pathological wound healing. *Arteriosclerosis, Thrombosis, and Vascular Biology*.

[24] Falanga V., Iwamoto S., Chartier M. (2007). Autologous bone marrow-derived cultured mesenchymal stem cells delivered in a fibrin spray accelerate healing in murine and human cutaneous wounds. *Tissue Engineering*.

[25] Fu X., Fang L., Li H., Li X., Cheng B., Sheng Z. (2007). Adipose tissue extract enhances skin wound healing. *Wound Repair and Regeneration*.

[26] Fu X., Fang L., Li X., Cheng B., Sheng Z. (2006). Enhanced wound-healing quality with bone marrow mesenchymal stem cells autografting after skin injury. *Wound Repair and Regeneration*.

[27] Gao W., Qiao X., Ma S., Cui L. (2011). Adipose-derived stem cells accelerate neovascularization in ischaemic diabetic skin flap via expression of hypoxia-inducible factor-1*α*. *Journal of Cellular and Molecular Medicine*.

[28] Gu J. H., Lee J. S., Kim D.-W., Yoon E.-S., Dhong E.-S. (2012). Neovascular potential of adipose-derived stromal cells (ASCs) from diabetic patients. *Wound Repair and Regeneration*.

[29] Hamou C., Callaghan M. J., Thangarajah H. (2009). Mesenchymal stem cells can participate in ischemic neovascularization. *Plastic and Reconstructive Surgery*.

[30] Heo S. C., Jeon E. S., Lee I. H., Kim H. S., Kim M. B., Kim J. H. (2011). Tumor necrosis factor-*α*-activated human adipose tissue-derived mesenchymal stem cells accelerate cutaneous wound healing through paracrine mechanisms. *Journal of Investigative Dermatology*.

[31] Hou C., Shen L., Huang Q. (2013). The effect of heme oxygenase-1 complexed with collagen on MSC performance in the treatment of diabetic ischemic ulcer. *Biomaterials*.

[32] Huang S., Lu G., Wu Y. (2012). Mesenchymal stem cells delivered in a microsphere-based engineered skin contribute to cutaneous wound healing and sweat gland repair. *Journal of Dermatological Science*.

[33] Javazon E. H., Keswani S. G., Badillo A. T. (2007). Enhanced epithelial gap closure and increased angiogenesis in wounds of diabetic mice treated with adult murine bone marrow stromal progenitor cells. *Wound Repair and Regeneration*.

[34] Kataoka K., Medina R. J., Kageyama T. (2003). Participation of adult mouse bone marrow cells in reconstitution of skin. *American Journal of Pathology*.

[35] Kim C. H., Lee J. H., Won J. H., Cho M. K. (2011). Mesenchymal stem cells improve wound healing in vivo via early activation of matrix metalloproteinase-9 and vascular endothelial growth factor. *Journal of Korean Medical Science*.

[36] Kim W.-S., Park B.-S., Sung J.-H. (2007). Wound healing effect of adipose-derived stem cells: a critical role of secretory factors on human dermal fibroblasts. *Journal of Dermatological Science*.

[37] Kim H., Choi K., Kweon O.-K., Kim W. H. (2012). Enhanced wound healing effect of canine adipose-derived mesenchymal stem cells with low-level laser therapy in athymic mice. *Journal of Dermatological Science*.

[38] Kwon D. S., Gao X., Liu Y. B. (2008). Treatment with bone marrow-derived stromal cells accelerates wound healing in diabetic rats. *International Wound Journal*.

[39] Lee S., Szilagyi E., Chen L. (2013). Activated mesenchymal stem cells increase wound tensile strength in aged mouse model via macrophages. *Journal of Surgical Research*.

[40] Lee E. Y., Xia Y., Kim W.-S. (2009). Hypoxia-enhanced wound-healing function of adipose-derived stem cells: Increase in stem cell proliferation and up-regulation of VEGF and bFGF. *Wound Repair and Regeneration*.

[41] Lee S. H., Lee J. H., Cho K. H. (2011). Effects of human adipose-derived stem cells on cutaneous wound healing in nude mice. *Annals of Dermatology*.

[42] Li H., Fu X., Ouyang Y., Cai C., Wang J., Sun T. (2006). Adult bone-marrow-derived mesenchymal stem cells contribute to wound healing of skin appendages. *Cell and Tissue Research*.

[43] Lim J. S., Yoo G. (2010). Effects of adipose-derived stromal cells and of their extract on wound healing in a mouse model. *Journal of Korean Medical Science*.

[44] Lin Y.-C., Grahovac T., Oh S. J., Ieraci M., Rubin J. P., Marra K. G. (2013). Evaluation of a multi-layer adipose-derived stem cell sheet in a full-thickness wound healing model. *Acta Biomaterialia*.

[45] Liu P., Deng Z., Han S. (2008). Tissue-engineered skin containing mesenchymal stem cells improves burn wounds. *Artificial Organs*.

[46] Liu S., Zhang H., Zhang X. (2011). Synergistic angiogenesis promoting effects of extracellular matrix scaffolds and adipose-derived stem cells during wound repair. *Tissue Engineering: Part A*.

[47] Maharlooei M. K., Bagheri M., Solhjou Z. (2011). Adipose tissue derived mesenchymal stem cell (AD-MSC) promotes skin wound healing in diabetic rats. *Diabetes Research and Clinical Practice*.

[48] Mansilla E., Spretz R., Larsen G. (2010). Outstanding survival and regeneration process by the use of intelligent acellular dermal matrices and mesenchymal stem cells in a burn pig model. *Transplantation Proceedings*.

[49] McFarlin K., Gao X., Liu Y. B. (2006). Bone marrow-derived mesenchymal stromal cells accelerate wound healing in the rat. *Wound Repair and Regeneration*.

[50] Nakagawa H., Akita S., Fukui M., Fujii T., Akino K. (2005). Human mesenchymal stem cells successfully improve skin-substitute wound healing. *British Journal of Dermatology*.

[51] Nambu M., Kishimoto S., Nakamura S. (2009). Accelerated wound healing in healing-impaired db/db mice by autologous adipose tissue-derived stromal cells combined with atelocollagen matrix. *Annals of Plastic Surgery*.

[52] Nambu M., Ishihara M., Nakamura S. (2007). Enhanced healing of mitomycin C-treated wounds in rats using inbred adipose tissue-derived stromal cells within an atelocollagen matrix. *Wound Repair and Regeneration*.

[53] Nie C., Yang D., Xu J., Si Z., Jin X., Zhang J. (2011). Locally administered adipose-derived stem cells accelerate wound healing through differentiation and vasculogenesis. *Cell Transplantation*.

[54] Rasulov M. F., Vasilenko V. T., Zaidenov V. A., Onishchenko N. A. (2006). Cell transplantation inhibits inflammatory reaction and stimulates repair processes in burn wound. *Bulletin of Experimental Biology and Medicine*.

[55] Rustad K. C., Wong V. W., Sorkin M. (2012). Enhancement of mesenchymal stem cell angiogenic capacity and stemness by a biomimetic hydrogel scaffold. *Biomaterials*.

[56] Sasaki M., Abe R., Fujita Y., Ando S., Inokuma D., Shimizu H. (2008). Mesenchymal stem cells are recruited into wounded skin and contribute to wound repair by transdifferentiation into multiple skin cell type. *The Journal of Immunology*.

[57] Sheng Z., Fu X., Cai S. (2009). Regeneration of functional sweat gland-like structures by transplanted differentiated bone marrow mesenchymal stem cells. *Wound Repair and Regeneration*.

[58] Shumakov V. I., Onishchenko N. A., Rasulov M. F., Krasheninnikov M. E., Zaidenov V. A. (2003). Mesenchymal bone marrow stem cells more effectively stimulate regeneration of deep burn wounds than embryonic fibroblasts. *Bulletin of Experimental Biology and Medicine*.

[59] Stoff A., Rivera A. A., Banerjee N. S. (2009). Promotion of incisional wound repair by human mesenchymal stem cell transplantation. *Experimental Dermatology*.

[60] Tian H., Lu Y., Shah S. P., Hong S. (2011). 14S,21R-dihydroxydocosahexaenoic acid remedies impaired healing and mesenchymal stem cell functions in diabetic wounds. *The Journal of Biological Chemistry*.

[61] Volk S. W., Radu A., Zhang L., Liechty K. W. (2007). Stromal progenitor cell therapy corrects the wound-healing defect in the ischemic rabbit ear model of chronic wound repair. *Wound Repair and Regeneration*.

[62] Wu Y., Chen L., Scott P. G., Tredget E. E. (2007). Mesenchymal stem cells enhance wound healing through differentiation and angiogenesis. *Stem Cells*.

[63] Yang M., Sheng L., Li H., Weng R., Li Q.-F. (2010). Improvement of the skin flap survival with the bone marrow-derived mononuclear cells transplantation in a rat model. *Microsurgery*.

[64] Yeum C. E., Park E. Y., Lee S.-B., Chun H.-J., Chae G.-T. (2013). Quantification of MSCs involved in wound healing: use of SIS to transfer MSCs to wound site and quantification of MSCs involved in skin wound healing. *Journal of Tissue Engineering and Regenerative Medicine*.

[65] Ko S. H., Nauta A., Wong V., Glotzbach J., Gurtner G. C., Longaker M. T. (2011). The role of stem cells in cutaneous wound healing: what do we really know?. *Plastic and Reconstructive Surgery*.

[66] Bourin P., Bunnell B. A., Casteilla L. (2013). Stromal cells from the adipose tissue-derived stromal vascular fraction and culture expanded adipose tissue-derived stromal/stem cells: a joint statement of the International Federation for Adipose Therapeutics and Science (IFATS) and the International Society for Cellular Therapy (ISCT). *Cytotherapy*.

[67] Levi B., Longaker M. T. (2011). Concise review: adipose-derived stromal cells for skeletal regenerative medicine. *Stem Cells*.

[68] Galiano R. D., Michaels V J., Dobryansky M., Levine J. P., Gurtner G. C. (2004). Quantitative and reproducible murine model of excisional wound healing. *Wound Repair and Regeneration*.

[69] Glotzbach J. P., Januszyk M., Vial I. N. (2011). An information theoretic, microfluidic-based single cell analysis permits identification of subpopulations among putatively homogeneous stem cells. *PLoS ONE*.

[70] Januszyk M., Sorkin M., Glotzbach J. P. (2014). Diabetes irreversibly depletes bone marrow-derived mesenchymal progenitor cell subpopulations. *Diabetes*.

[71] Chung M. T., Liu C., Hyun J. S. (2013). CD90 (Thy-1)-positive selection enhances osteogenic capacity of human adipose-derived stromal cells. *Tissue Engineering Part A*.

[72] Mansilla E., Aquino V. D., Roque G., Tau J. M., MacEira A. (2012). Time and regeneration in burns treatment: heading into the first worldwide clinical trial with cadaveric mesenchymal stem cells. *Burns*.

[73] Dabiri G., Heiner D., Falanga V. (2013). The emerging use of bone marrow-derived mesenchymal stem cells in the treatment of human chronic wounds. *Expert Opinion on Emerging Drugs*.

[74] Wong V. W., Rustad K. C., Galvez M. G. (2011). Engineered pullulan-collagen composite dermal hydrogels improve early cutaneous wound healing. *Tissue Engineering Part A*.

[75] Clover A. J., Kumar A. H., Isakson M. (2015). Allogeneic mesenchymal stem cells, but not culture modified monocytes, improve burn wound healing. *Burns*.

[76] Wong V. W., Gurtner G. C., Longaker M. T. (2013). Wound healing: a paradigm for regeneration. *Mayo Clinic Proceedings*.

[77] McArdle A., Lo D. D., Hyun J. S. (2013). Discussion: a report of the ASPS task force on regenerative medicine: opportunities for plastic surgery. *Plastic and Reconstructive Surgery*.

[78] Whelan D., Caplice N. M., Clover A. J. (2014). Fibrin as a delivery system in wound healing tissue engineering applications. *Journal of Controlled Release*.

[79] Rasulov M. F., Vasilchenkov A. V., Onishchenko N. A. (2005). First experience of the use bone marrow mesenchymal stem cells for the treatment of a patient with deep skin burns. *Bulletin of Experimental Biology and Medicine*.

[80] Nambu M., Kishimoto S., Nakamura S. (2009). Accelerated wound healing in healing-impaired db/db mice by autologous adipose tissue-derived stromal cells combined with atelocollagen matrix. *Annals of Plastic Surgery*.

[81] Sasaki M., Abe R., Fujita Y., Ando S., Inokuma D., Shimizu H. (2008). Mesenchymal stem cells are recruited into wounded skin and contribute to wound repair by transdifferentiation into multiple skin cell type. *Journal of Immunology*.

[82] Nauta A., Seidel C., Deveza L. (2013). Adipose-derived stromal cells overexpressing vascular endothelial growth factor accelerate mouse excisional wound healing. *Molecular Therapy*.

[83] Badiavas E. V., Falanga V. (2003). Treatment of chronic wounds with bone marrow-derived cells. *Archives of Dermatology*.

[84] Dash N. R., Dash S. N., Routray P., Mohapatra S., Mohapatra P. C. (2009). Targeting nonhealing ulcers of lower extremity in human through autologous bone marrow-derived mesenchymal stem cells. *Rejuvenation Research*.

[85] Yoshikawa T., Mitsuno H., Nonaka I. (2008). Wound therapy by marrow mesenchymal cell transplantation. *Plastic and Reconstructive Surgery*.

[86] Rasulov M. F., Sevast'ianov V. I., Egorova V. A. (2005). A comparative study of the dynamics of deep burns healing in using medullary allogenic fibroblast-like mesenchymal stem cells from bone marrow immobilized on biodegrading membranes or taken from cultural plastic. *Patologicheskaia Fiziologiia i Eksperimental'naia Terapiia*.

[87] Yoshikawa T., Mitsuno H., Nonaka I. (2008). Wound therapy by marrow mesenchymal cell transplantation. *Plastic and Reconstructive Surgery*.

[88] Siev-Ner I. (2012). *Safety Study of Stem Cells Treatment in Diabetic Foot Ulcers*.

[89] Falanga V. (2012). *Study of the Effectiveness of Autologous Bone Marrow-Derived Mesenchymal Stem Cells in Fibrin to Treat Chronic Wounds*.

[90] Baksh D., Song L., Tuan R. S. (2004). Adult mesenchymal stem cells: characterization, differentiation, and application in cell and gene therapy. *Journal of Cellular and Molecular Medicine*.

[91] Ma K., Laco F., Ramakrishna S., Liao S., Chan C. K. (2009). Differentiation of bone marrow-derived mesenchymal stem cells into multi-layered epidermis-like cells in 3D organotypic coculture. *Biomaterials*.

[92] Li H. H., Fu X. (2012). Mechanisms of action of mesenchymal stem cells in cutaneous wound repair and regeneration. *Cell and Tissue Research*.

[93] Galindo L. T., Filippo T. R. M., Patricia S. (2011). Mesenchymal stem cell therapy modulates the inflammatory response in experimental traumatic brain injury. *Neurology Research International*.

[94] Yoon B. S., Moon J.-H., Jun E. K. (2010). Secretory profiles and wound healing effects of human amniotic fluid-derived mesenchymal stem cells. *Stem Cells and Development*.

[95] Mansilla E., Marin G. H., Sturla F. (2005). Human mesenchymal stem cells are tolerized by mice and improve skin and spinal cord injuries. *Transplantation Proceedings*.

[96] Senarath-Yapa K., McArdle A., Renda A., Longaker M. T., Quarto N. (2014). Adipose-derived stem cells: a review of signaling networks governing cell fate and regenerative potential in the context of craniofacial and long bone skeletal repair. *International Journal of Molecular Sciences*.

[97] Cai S., Pan Y., Han B., Sun T.-Z., Sheng Z.-Y., Fu X.-B. (2011). Transplantation of human bone marrow-derived mesenchymal stem cells transfected with ectodysplasin for regeneration of sweat glands. *Chinese Medical Journal*.

[98] Fu X.-B., Fang L.-J., Wang Y.-X., Sun T.-Z., Cheng B. (2004). Enhancing the repair quality of skin injury on porcine after autografting with the bone marrow mesenchymal stem cells. *Zhonghua Yi Xue Za Zhi*.

[99] Zhang C., Ha X.-Q., Liu Y., Jin L.-J., Lü T.-D. (2008). Bone marrow mesenchymal stem cells transfected with hepatocyte growth factor gene for repair of burn wound after allotransplantation. *Journal of Clinical Rehabilitative Tissue Engineering Research*.

[100] Sheng Z., Fu X., Cai S. (2009). Regeneration of functional sweat gland-like structures by transplanted differentiated bone marrow mesenchymal stem cells. *Wound Repair and Regeneration*.

[101] Liu H., Li X.-Q., Chen W.-P., Xiang H.-X., Hu J., Zhang X.-W. (2009). Construction of tissue engineered skin by collagen with bone mesenchymal stem cells transfected with angiotensin I for repairing wound surface in scald models. *Journal of Clinical Rehabilitative Tissue Engineering Research*.

[102] McArdle A., Senarath-Yapa K., Walmsley G. G. (2014). The role of stem cells in aesthetic surgery: fact or fiction?. *Plastic and Reconstructive Surgery*.

